# COVID-19 pandemia and inherited cardiomyopathies and channelopathies: a short term and long term perspective

**DOI:** 10.1186/s13023-020-01444-2

**Published:** 2020-06-22

**Authors:** Giuseppe Limongelli, Lia Crotti

**Affiliations:** 1 Inherited and Rare Cardiovascular Disease Clinic, Department of Translational Medical Sciences, University of Campania “Luigi Vanvitelli”, Via L. Bianchi 1 c/o Monaldi Hospital, AORN Colli, Naples, Italy; 2 Institute of Cardiovascular Sciences, University College of London and St. Bartholomew’s Hospital, London, UK; 3 Centro coordinamento malattie rare regione campania - via L. bianchi 1 c/o monaldi hospital - ao colli - Naples, Naples, Italy; 4 European Reference Network for Rare and Complex Diseases of the Heart, http://guardheart.ern-net.eu; 5 Istituto Auxologico Italiano, IRCCS, Department of Cardiovascular, Neural and Metabolic 5. Sciences, San Luca Hospital, Milan, Italy; 6 Istituto Auxologico Italiano, IRCCS, Center for Cardiac Arrhythmias of Genetic Origin, Laboratory of Cardiovascular Genetics, Milan, Italy; 7 Department of Medicine and Surgery, University of Milano-Bicocca, Milan, Italy

**Keywords:** Inherited and rare heart disease, Cardiomyopathies, Channellopathies, COVID-19

## Abstract

Inherited heart disease represent a very heterogenous group of cardiac disorders, characterized by inherited, acquired, and often rare disorders affecting the heart muscle (cardiomyopathies) or the cardiac electrical system (ion channel disease). They are often familial diseases, and are among the leading cause of juvenile sudden death and heart failure. The aim of this paper is to give a perspective on how to run a clinical service during an epidemic or pandemic emergency and to describe the potential COVID-19 associated risks for patients affected by inherited heart diseases.

## Introduction

The severe acute respiratory syndrome coronavirus-2 (SARS-Cov-2) has been recently declared as pandemic by WHO, emerging as a global threat due to the high transmission rate of the virus. The infection may have a wide range of clinical manifestation from no symptoms to interstitial pneumonia, acute respiratory distress and/or systemic disease. Moreover, data show that pre-existing cardiovascular diseases may favor more severe clinical manifestations [1], and conversely SARS-CoV-2 may cause myocardial injury [2]. The exponential need of hospitalization and intensive care due to COVID-19 infections thoroughly changed priorities of healthcare systems around the world. Hospitals were in part or completely dedicated to Covid-19 patients and therefore routine management of patients with inherited channelopathies and cardiomyopathies was suspended in most hospitals.

Inherited and rare heart diseases represent a very heterogenous group of congenital, inherited or acquired disorders affecting the heart muscle (cardiomyopathies) or the electrical system (channelopathies). They are often familial diseases, and are among the leading cause of juvenile sudden death and heart failure. The aim of this paper is to give a perspective on how to run an inherited and rare disease clinical service during an epidemic or pandemic emergency and to describe the potential COVID-19 associated risks for patients affected by these diseases.

## What are the risks associated with Covid-19 infection for patients with inherited cardiomyopathies and channelopathies?

Channelopathies are a group of genetically transmitted heart diseases, in which the heart is mainly structural normal, but there is an increased risk of life-threatening arrhythmias.

In Long QT Syndrome (LQTS) [3], QT prolonging drugs should be avoided as they could favor torsades de pointes (TdP) and sudden cardiac death [4]. In case of COVID-19 infection most of the drugs currently used (Table 1) can prolong QT interval [5, 6] and some of them may cause diarrhea, favoring hypokalemia, another factor increasing arrhythmia risk [3]. Furthermore, diarrhea is one of the clinical manifestation of COVID-19 infection (Table 2) [1]. Therefore, specific caution should be suggested in LQTS patients to avoid infection and whenever they get it is, it is clearly dangerous an in house management with drugs in the absence of an adequate QT monitoring. Whenever an in-hospital admission is needed, a careful QT monitoring and a telemetric system should be used. A careful balance of pros and cons should guide the decision to discontinue therapy in case of important QT prolongation. Furthermore, in the absence of clear benefit and safety data from well designed, randomized, controlled clinical trials, therapies associated with greater QT prolongation and arrhythmic risk, as hydroxychloroquine/chloriquine particularly if in association with macrolide (azitromicin), should be avoided. Mandatory is going on with beta-blocker therapy and keep potassium level above 4 mEq/l with potassium supplements.

**Table 1:** Current medications used in Covid-19 infection and potential side effects in cardiomyopathies (CMP) and ion channel diseases (ICD)

DRUG	SIDE EFFECTS	USE in CMP and ICD
H**ydroxychloroquine**^a^	QT prolongation (drug effect plus CYP3A4 inhibition)	Potentially harmful in LQTs, HCM or other CMPs associated with LQT, acquired QT status Caution in: hypokaliemia status, severe hypoglicemia, renal or epatic failure; digoxin, antiepilectics or ciclosporin therapy; patients with G6PDH and porfiria
**Azitromicin**^a^	QT prolongation (drug effect plus mild CYP3A4 inhibition)	Potentially harmful in LQTs, HCM or other structural disease associated with LQT, acquired QT status
**Ritonavir**	QT prolongation (CYP3A4 inhibition) Bradyarrhythmias/AV blocks Hypertension, angioedema, maculopapular rash, respiratory tract infection, peripheral neuropathy, hypercholesterolemia, hypertriglyceridemia, increased glucose, increased uric acid, increased transaminases and creatine kinase, decreased CrCl, neutropenia, anemia Rare: ketoacidosis, insulin resistance, anorexia, hyperlactatemia, rhabdomyolysis	Potentially harmful in LQTs, HCM or other structural disease associated with LQT, acquired QT status Caution in patients with congenital, inherited (i.e. SCN5A), or structural (i.e. Lamin A/C, desmin, mitchocondrial) AV blocks Caution in patients with previous renal and hepatic diseases and/or previous peripheral neuropathy (i.e. Amyloidosis, Fabry disease), hypertension, familial hypercolesterolemia, uncompensated DM, mitochondrial or metabolic disorders, syndromes associated with anemia or neutropenia (i.e. Barth synfrome) Caution in patients taking sildenafil (i.e. pulmonary hypertension), sinvastatin, amiodaron, midazolam
**Lopinavir**	QT prolongation (CYP3A4 inhibition) Bradyarrhythmias/AV blocks Hypertension, angioedema, maculopapular rash, respiratory tract infection, peripheral neuropathy, hypercholesterolemia, hypertriglyceridemia, increased glucose, increased uric acid, increased transaminases and creatine kinase, decreased CrCl, neutropenia, anemia Rare: ketoacidosis, insulin resistance, anorexia, hyperlactatemia, rhabdomyolysis	Potentially harmful in LQTs, HCM or other structural disease associated with LQT, acquired QT status Caution in patients with congenital, inherited (i.e. SCN5A), or structural (i.e. Lamin A/C, desmin, mitchocondrial) AV blocks Caution in patients with previous renal and hepatic diseases and/or previous peripheral neuropathy (i.e. Amyloidosis, Fabry disease), hypertension, familial hypercolesterolemia, uncompensated DM, mitochondrial or metabolic disorders, syndromes associated with anemia or neutropenia (i.e. Barth synfrome) Caution in patients taking sildenafil (i.e. pulmonary hypertension), sinvastatin, amiodaron, midazolam
**Remdesivir**	No definite effect on cardiac electrical activity	Liver enzyme increase
**Tocilizumab**	No definite effect on cardiac electrical activity Drug idiosyncrasy, hypertension, hypercolesterolemia, respiratory tract or other infections, increased transaminase, reduced CrCl,	Caution in patients with previous renal and hepatic diseases, hypertension, familial hypercolesterolemia Caution in patients taking other immunosoppressive drugs (i.e. cortisone, ciclosporin), simvastatin/atorvastatin, amlodipin, teofillin, warfarin, temazepam
**Low molecular weight Eparin**	No definite effect on cardiac electrical activity Uncontrolled bleeding Heparin induced thrombocytopenia Elevated liver enzymes	Caution in patients with splenomegaly status (i.e. Gaucher disease, amyloidosis, sarcoidosis) Caution in patients with previous thrombocytopenia or coagulation factors deficits (i.e. Rasopathies: Noonan syndrome) Caution in patients with severe renal and hepatic diseases Caution in patients taking high dose diuretics, captopril, abciximab, clopidogrel, digoxin

^a^*In absence of clear benefit and safety data from well designed, randomized, controlled clinical trials, the WHO and many national authorities have issued specific warnings for the use of hydroxychloroquine, particularly in association with azitromicin (*https://www.who.int/emergencies/diseases/novel-coronavirus-2019/coronavirus-disease-answers?query=hydroxychloroquine*)*

**Table 2:** Symptoms and sign of Covid-19 infection and management in cardiomyopathies (CMP) and ion channel diseases (ICD). HFpEF: heart failure with preserved ejection fraction; HFrEF: heart failure with reduced ejection fraction

SYMPTOM	CLINICAL CONTEXT	MANAGEMENT
**Fever**	*anamnesis: previous contact with Covid19+, onset, and progression, degree (>or < 37.5°), association with other symptoms*	*Hydratation (according to clinical status) and paracetamol* *ECG monitoring in Brugada Syndrome. Consider hospitalization in high risk patients (BS type 1 with no ICD; previous syncope; persistent fever, with no response to paracetamol)*
**Cough**	*anamnesis: previous contact with Covid19+, type of cough (productive or dry), previous or recent onset, association with other symptoms*	Teleconsultation ProBNP or BNP (suspect of new onset heart failure), when possible Consider hospitalization in high risk patients (emergency; end stage cardiomyopathies; high suspect of HFREF/HFPEF)
**Dyspnoea**	*anamnesis: previous contact with Covid19+, type and degree, previous or recent onset, association with other symptoms*	Teleconsultation ProBNP or BNP (suspect of new onset heart failure), when possible Consider hospitalization in high risk patients (emergency; end stage cardiomyopathies; high suspect of HFREF/HFPEF)
**Fatigue**	*anamnesis: previous contact with Covid19+, type (*i.e. *myalgia, cramps, etc) and degree, previous of recent onset, association with other symptoms*	Teleconsultation When useful, suggest potassium/magnesium supplementation ProBNP or BNP (suspect of new onset heart failure), when possible Consider hospitalization in high risk patients (emergency; end stage cardiomyopathies; high suspect of HFREF/HFPEF)
**Diarrhea**	*anamnesis: previous contact with Covid19+, association with other symptoms*	Teleconsultation Hydratation (according to clinical status) Risk of hypokaliemia particularly dangerous in patients with prolonged QT (LQTs, drugs, HCM) Potassium/magnesium supplementation

Very recently, CredibleMeds launched an important new decision support program to help clinicians manage the risk of QT prolonging medications when treating patients with COVID-19. Accessing at MedSafety Scan® (https://medsafetyscan.org) and entering each patient’s clinical risk factors and their drugs, the program quickly reports if any of the medicines are on the QTdrugs lists and calculates the patient’s QTscore for risk of QT prolongation and torsades.

In Short QT Syndrome (SQTS) [3], there is no particular concern related to COVID-19 infection, the only precaution should be avoid hypokalemia that is a pro-arrhythmic factor. The same apply to catecholaminergic polymorphic ventricular tachycardia (CPVT), in which an additional risk could be the use of alpha or beta adrenergic mimetic drugs in case a hemodynamic support is needed [6].

For Brugada Syndrome fever related to infection is the real danger and a more careful and tailored evaluation of criteria for admission to Hospital should be applied [6].

Cardiomyopathies (CMPs) represent an heterogeneous group of inherited disease of the heart muscle, characterized by different phenotypes (hypertrophic, HCM; dilated, DCM; arrhythmogenic, AC; restrictive, RCM) and dysfunction (diastolic and/or systolic), with an increased risk of life-threatening arrhythmias and heart failure with preserved or reduced ejection fraction (HFpEF or HFrEF).

It is now clear that Covid-19 infection has a wide spectrum of presentation and complications, related to the virus itself or secondary to the inflammatory and immune response [7]. Myocardial injury, evidenced by elevated high-sensitivity troponin levels, is more frequently observed in severe cases, and it is associated with the higher inflammatory burden that can induce vascular inflammation, myocarditis, and cardiac arrhythmias [8].

Covid-19 infection in patients with CMPs represent a concrete risk of worsening patient clinical status, particularly in those who experienced previous HF events or with end stage disease. Moreover, the combination of hypokalemia and prolonged QT exponentially increases the risk of arrhythmias. Hospitalization should be considered, after teleconsultation, in patients with moderate-high risk of disease worsening.

Also, there is a specific risk related to CMP phenotype (i.e. the risk of dehydration can be much higher in patients with obstructive vs non obstructive HCM) [9] or etiology (increase risk of metabolic decompensation or lactic acidosis in patients with metabolic and mitochondrial CMPs and myopathies) (Table 3) [10, 11].

**Table 3:** Management of cardiomyopathies (CMP) and ion channel diseases (ICD), according to phenotype and etiology

INHERITED CARDIAC DISEASE	MANAGEMENT
**Hypertrophic Cardiomyopathy (HCM)**	Avoid dehydration in obstructive HCM (fever, diarrhea) QT monitoring, especially in patients on dysopiramide (Covid 19 therapies)
**Dilated Cardiomyopathy (DCM)**	Balance fluid and electrolyte intake according to clinical status (fever, diarrhea) QT monitoring (Covid 19 therapies) Exclude new onset arrhythmias (palpitations) HF (dyspnoea) Do not stop ACE-i and ARBs (consider that ACE-i worse cough) Hospital admission if progressive symptoms
**Arrhythmogenic Cardiomyopathy (AC)**	QT monitoring, especially in patient on sotalol (Covid 19 therapies) Exclude new onset arrhythmias (palpitations) HF (dyspnoea) Hospital admission if progressive symptoms
**Restrictive Cardiomyopathy (RCM)**	As DCM
**Noncompaction (NC)**	As DCM
**Myocarditis/Inflammatory Cardiomyopathy**	As DCM EMB should be considered to exclude specific treatment
**Childhood Cardiomyopathies**	Avoid dehydration and balance fluid and electrolyte intake according to clinical status (fever, diarrhea) No specific Covid 19 treatment required in most cases
**Genetic Syndromes**	Increased risk of bleeding in patients with Noonan syndrome/rasopathies and coagulation factors deficits (Covid 19 therapies)
**Neuromucular Disease**	ECG monitoring: bradyarrhythmias, AV blocks, prolonged QT (Covid 19 therapies)
**Mitochondrial Disease**	Lactic acidosis crisis, hypoglycemia, fatigue, rabdomyolisis (fever, diarrhea, Covid 19 therapies) ECG monitoring: bradyarrhythmias, AV blocks, prolonged QT (Covid 19 therapies) Hospital admission if progressive symptoms/crisis
**Glycogen Storage Disease**	Metabolic crisis, fatigue, cramps (fever, diarrhea, Covid 19 therapies) ECG monitoring: bradyarrhythmias, AV blocks, prolonged QT (Covid 19 therapies)
**Lysosomal Storage Disease**	Symptoms worsening (i.e. Fabry crisis; fever, diarrhea) ECG monitoring: bradyarrhythmias, AV blocks, prolonged QT (Covid 19 therapies) Consider “home therapy” for enzyme replacement therapies (ERTs)
**Amyloidoses**	ECG monitoring: bradyarrhythmias, AV blocks, prolonged QT (Covid 19 therapies) Clinical status may worsen (Covid 19 therapy) Consider to stop specific therapy protocol in AL Tafamidis (TTR) can worsen cough
**Long QT Syndrome (LQTS)**	QT prolungation (Covid 19 therapy) Consider hospitalization in high risk patients
**Short QT Syndrome (SQTS)**	QT prolungation (Covid 19 therapy)
**Brugada Syndrome (BS)**	Type 1 BS pattern (fever) Consider hospitalization in high risk patients
**Catecholaminergic Polymorphic Ventricualr Tachicardia (CPVT)**	Epinephrine in patients who require haemodynamic support is proarrhythmic Consider hospitalization in high risk patients
**SCN5A Disease**	Type 1 BS pattern (fever) Risk of bradyarrhythmias/AV blocks (Covid 19 therapy) Consider hospitalization in high risk patients

## How to run an inherited heart disease clinic during COVID-19 pandemia

**Fig. 1:**
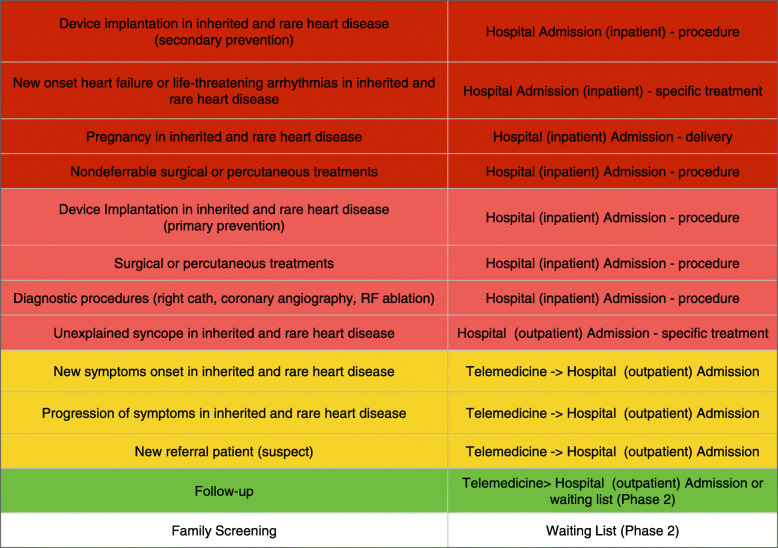
how to run an inherited heart disease clinic during covid-19 pandemia. List of priorities

The big challenge during the pandemic is the “list of priorities” that the healthcare system need to redesign to face the emergency. The healthcare systems of the vast majority of western countries have been built to treat cardiovascular and neurological emergencies, and to treat chronic patients with different disease. In this latter, inherited and rare disease represent a wide spectrum of cardiovascular disease generally diagnosed and managed by a multidisciplinary setting of expert physicians. As in Fig. 1, we designed a 5 levels list of priorities in these patients: 1. Level 1: all the patients with severe, new onset symptoms (heart failure, HF) or life-threatening arrhythmias requiring immediate treatment; pregnancy management; nondeferrable surgical or percutaneous treatments; 2. Level 2: urgent diagnostic, devices, percutaneous, or surgical procedures; 3. Level 3: new symptoms onset or progressive symptomatology; new referral; 4. Level 4: patients follow-up; 5. Level 5: family screening. The first 2 levels deserve immediate or urgent hospitalization for procedures or nondeferrable treatments. This should follow a “non COVID-19 pathway”, with dedicated spaces and equipment. Before admission, or at admission for very urgent condition, a COVID-19 PCR nasal and throat swab should be performed in all patients. The 3rd and 4rth level can be managed with teleconsultation, eventually followed by outpatient clinic admission. Family screening can be planned for the post-emergency phase (phase 2).

Phase 2 is the most challenging phase. Since phase 1 had a “leopard spot pattern” of presentation, progression and severity in different regions and countries, timing and specific measure for phase 2 can be different and region/country specific. Nevertheless, there is a common goal that should be pursued, i.e. population safety and the avoidance of virus spreading. This could be obtained considering anyone as potentially positive. Therefore, for everyone, social distancing and the use of facial masks should be mandatory and adequate DPI should be worn by healthcare personnel, to avoid not only that they get ill but that they could become carriers of the infection. Population control with the use of COVID-19 IgG/IgM (and, in selected cases, COVID-19 PCR nasal and throat swab) associated with smart-phone technologies, could be of great help to guide a safer phase 2.

In term of organization of the inherited heart disease clinic, 4 main points should be considered:

1. a priority based approach: different levels of intervention (inhospital admission, outpatient clinic evaluation, teleconsulting) should be created according to the clinical status and specific needs of different patients. The same applies for hospital based and/or “home therapies” (as for patients with CMPs and lysosomal storage disorders).
2. triage step: a team of doctors, nurses, and trainees, should be in charge for the triage step, i.e. to define a new agenda of the outpatient clinic which is priority based.
3. teleconsultation/telemedicine: before admission, the triage team can preventively contact by teleconsulting/telemedicine platforms patients and their families, to collect past and recent clinical history (including, any symptoms or contact with infected individuals).
4. safety procedures: an online booking and payment should be provided by the hospital/academic organization. The admission in the outpatients clinic will be preordered to avoid crowded waiting rooms and favor social distancing, as much as possible. Adult patients will be invited to come alone (with very few exceptions) to clinic, while no more than 1 person will be allowed to come with children. At admission, body temperature will be measured, and surgical masks should be worn by the all patients.

## Conclusions

Patients with inherited CMPs and channelopaties are at potential higher risk during a COVID-19 infection and disease-specific recommendation and precaution should be employed. There are risks associated with the infection itself, which can cause interstitial pneumonia, but could also have a cardiac and/or systemic involvement, and risks related to COVID-19 treatment. Moreover, little is known about medium- and long-term consequences of this infection that could theoretically favor CMPs secondary to acute cardiac injuries (i.e. myocarditis, acute coronary syndromes) or to pulmonary sequelae that may favor chronic pulmonary heart.

Inherited and rare disease clinical services should adjust their way of managing patients during an epidemic or pandemic emergency and we proposed possible schemes. A teleconsulting/telemedicine approach should be favorably deemed during Covid-19 emergency, and should strongly support accomplishment of phase 2.

## Data Availability

N/A

## Abbreviations

AC: Arrhythmogenic cardiomyopathy
CMPs: Cardiomyopathies
CPVT: Catecholaminergic polymorphic ventricular tachycardia
DCM: Dilated cardiomyopathy
HFpEF: Heart failure with preserved ejection fraction
HFrEF: Heart failure with reduced ejection fraction
HCM: Hypertrophic cardiomyopathy
LQTS: Long QT Syndrome
RCM: Restrictive cardiomyopathy
SARS-Cov-2: Severe acute respiratory syndrome coronavirus-2
SQTS: Short QT Syndrome
